# A Timely Exploratory Study of Young Adults’ Psychosexual Responses to the UK’s New Age Verification Law on Online Pornography

**DOI:** 10.1007/s10964-026-02397-8

**Published:** 2026-08-03

**Authors:** Yishu Li, Roger Ingham, Heather Armstrong

**Affiliations:** Centre for Sexual Health Research, School of Psychology, University of Southampton, Southampton, UK

**Keywords:** Age verification law, psychosexual well-being, mixed method, young adult, digital media, online pornography

## Abstract

Age-verification policies for online pornography aim to protect minors, yet their implications for legal-age young adults remain underexamined. This mixed-methods study examined UK young adults aged 18–25 who reported pornography use in the past year (*N* = 623, Mage = 21.40, SD = 2.31; 67.3% female). Participants completed measures of pornography use contexts, content preferences, and sexual shame, and answered open-ended questions about the UK age-verification requirements. Descriptive quantitative findings characterized participants’ use contexts, preferences, and sexual shame. Qualitative accounts described varied psychological experiences, including shame, anxiety, confidence, calm, and minimal emotional impact. Participants often interpreted age verification as making pornography access feel less anonymous and more identity-linked, raising concerns about surveillance, moral stigma, and data vulnerability. Reported access practices included platform avoidance, alternative online spaces, reduced use, and VPN-based circumvention. The findings highlight the need to consider privacy, stigma, trust, accountability, and access displacement when evaluating age-verification requirements for legal-age users.

**Supplementary Information:**

The online version contains supplementary material available at https://doi.org/10.1007/s10964-026-02397-8.

## Introduction

The visibility and easy availability of online pornography have raised concerns about users’ sexual socialization, well-being, and digital safety (Paulus et al., 2024). In response, a growing number of countries have advanced regulatory approaches aimed at reducing minors’ access to online pornography, most commonly through age-verification or age-assurance requirements on commercial pornography services (Lang et al., 2026 (United Status); Thurman et al., 2022 (France); Turvey et al., 2024 (Australia); Woodley et al., 2025 (Australia). Comparative policy analyses indicate that such measures are increasingly framed around platform responsibility and technological solutions, while public perceptions remain divided with regard to their effectiveness, privacy implications, and broader social consequences (Jarvie & Renaud, 2024 (UK). In the UK, similar measures have only recently been introduced, with a new age-verification law for online pornography coming into force and creating a period of regulatory transition (Ofcom, 2025). Although age-verification policies are primarily designed to restrict minors’ access, their implications for young adults, who are legally permitted users but remain in a phase of consolidating sexual identity, norms, and relational practices, are less well understood. Drawing on stigma and surveillance perspectives, this study examines how UK young adults describe pornography access, privacy, visibility, disclosure, and sexual shame within this age-verification context.

### Young Adults’ Pornography Use and Sexual Shame

Much of the public and policy debate has focused on adolescents, reflecting concerns about how access to sexually explicit material may intersect with sexual socialization and digital safety during a period of rapid developmental change (Andrie et al., 2021; Pathmendra et al., 2023; Paulus et al., 2024). Reviews of adolescent-focused research suggest that pornography use can be associated with sexual scripts, body-related perceptions, and emerging norms around intimacy and consent (Paulus et al., 2024; Peter & Valkenburg, 2016). This body of work provides important insight into developmental timing and vulnerability, but its findings cannot be assumed to generalize directly to young adults. Distinguishing these bodies of evidence helps to avoid treating findings from adolescent samples as interchangeable with those from young adults^1^, the population examined in the present study. At the same time, emerging debates around the regulation of online pornography suggest that such interventions may have broader implications beyond restricting access alone. For example, concerns have been raised that age-verification requirements may alter how pornography use is experienced by making it feel less anonymous and more closely tied to personal identity or data disclosure, particularly in contexts where users are required to provide sensitive information or interact with verification systems (Blake, 2019). Under these conditions, individuals may describe and subjectively interpret pornography access not only in terms of availability, but also in terms of anonymity, disclosure, concealment, and perceived data vulnerability.

Pornography use in young adults is often discussed as more varied in purpose and context, including its potential role as an informal source of sexual learning, where formal sex education is perceived as limited (Litsou et al., 2021). Evidence from young adult samples further highlights heterogeneity in both use contexts and content, and suggests associations with sexual scripts and norms, as well as with exposure to more extreme or degrading material (Dwulit and Rzymski 2019; Fernández-Ruiz et al. 2023; Li et al. 2025a). Contemporary research also emphasizes that pornography use is not a single behavior but a set of practices that occur across multiple relational and interpersonal contexts (Kohut et al., 2021; Mardegan, 2024). Young adults may use pornography privately, transparently, or jointly with partners, reflecting different degrees of openness and relational negotiation (Huntington et al., 2021; Li et al., 2026). Moreover, variation is also evident in the types of content young adults prefer. Surveys and qualitative studies indicate preferences ranging from mainstream depictions (e.g., vaginal intercourse, oral sex) to more specific genres that reflect individual curiosity, exploration, or particular arousal patterns (Dwulit & Rzymski, 2019; Fernández-Ruiz et al., 2023). A recent systematic review reported gendered patterns in content, with both men and women more likely to view male-centric pornography than female-centric material and men more frequently endorsing degrading, extreme, or taboo genres (Li et al. 2025a). Content preferences can also contribute positively by offering opportunities for exploration and informal sexual learning (Litsou et al., 2021), yet may also reinforce unrealistic sexual scripts, appearance ideals, or expectations about partners, particularly where violent or degrading material is normalized (Government Equalities Office, 2021; Li et al. 2025a). Together, pornography use contexts and content preferences help characterize the range of pornography-related experiences that young adults may bring to the age-verification context.

Pornography use may also carry social and moral meanings beyond just the act of viewing. Stigma theory conceptualizes stigma as a social process through which behaviors or identities become labelled, negatively stereotyped, and positioned as discreditable and discrediting within a given context (Andersen et al., 2022). Recent UK-based qualitative research shows that pornography use may be experienced as a private or stigmatised practice, with users managing potential judgement through concealment, distancing, and other stigma-management strategies (Macdowall et al., 2025). This perspective is relevant to participants’ accounts of shame, discomfort, self-perception, and perceived judgement in relation to age verification.

A growing body of research highlights the complex ways in which pornography use is associated with sexual shame, which has been consistently related to distress, internalized stigma, and reduced sexual wellbeing (Floyd et al., 2022; Sævik & Konijnenberg, 2023). In recent UK-based work, these dynamics appear to be influenced by both attitudes and patterns of use. This qualitative research shows that pornography use is often experienced as a stigmatized and “private” practice, with users describing efforts to conceal their viewing, distance themselves from “problematic” consumers, and manage feelings of embarrassment, guilt, or “silent” shame, particularly among women (Macdowall et al., 2025). Survey data with young women similarly indicate that more negative attitudes towards pornography use are associated with greater sexual shame, whereas more positive attitudes are linked to higher sexual satisfaction and comfort with sex, partly via more open sexual communication with partners (Li et al. 2025b). Findings on young adults’ use patterns further suggest that secret pornography use is associated with higher relational sexual shame, whereas shared use is associated with lower relational and internalized shame (Li et al., 2026). However, little is known about how young adults themselves describe shame, discomfort, emotional responses, or self-perception in relation to age-verification requirements.

### The New Age-Verification Law for Online Pornography in the UK

In 2025, the UK brought into force new age-verification requirements for online pornography under the Online Safety Act 2023. From 25 July, commercial pornography websites and other services hosting pornographic content were legally required to implement “highly effective” age checks to prevent children from accessing sexually explicit material (HM Government, 2023; Ofcom, 2025). These provisions introduced new statutory obligations for age verification on commercial pornography services, thereby reshaping the legal framework governing access to online sexual content. Legal research has detailed how the Online Safety Act 2023 defines the scope of these obligations and positions age verification as a central regulatory mechanism for controlling access to pornographic content (McGlynn et al., 2024). Policy-oriented commentary has examined how age-verification measures are embedded within broader approaches to child online safety, including expectations around platform responsibility and the management of digital risk (Livingstone, 2025). At the same time, comparative policy research conducted prior to implementation highlighted ongoing debate and uncertainty regarding the likely effectiveness of such measures, as well as concerns about privacy and wider social consequences (Jarvie & Renaud, 2024).

These privacy concerns can be understood through a surveillance perspective. Surveillance theory conceptualizes surveillance as systematic attention to personal details, increasingly involving digital observation, sorting, identification, and dataveillance (Lyon, 2022). In the context of online pornography, age verification may therefore be experienced not only as an access-control mechanism, but also as a process that links sexual content access to identity checks, personal data, or third-party verification systems. Recent work on online age verification similarly highlights public concerns about privacy-invasive implementation, supplier responsibility, and uncertainty about effectiveness (Jarvie & Renaud, 2024). This perspective is relevant to participants’ accounts of privacy, perceived monitoring, data vulnerability, and alternative access pathways within the age-verification context.

## The Current Study

Despite growing regulatory and policy attention to online pornography age verification, empirical research on legally permitted young adults’ experiences remains limited. In particular, less is known about how young adults make sense of age-verification requirements in relation to pornography access, privacy, emotional responses, bypassing strategies, and policy legitimacy. To address this gap, the present study examined UK young adults’ pornography-related experiences and perceptions following the introduction of age-verification requirements for online pornography. Descriptive quantitative data characterized participants’ pornography use contexts, content preferences, and sexual shame, while open-ended responses captured participants’ own accounts of the emotional, behavioral, and attitudinal meanings of age-verification requirements. The central research question concerned how young adults described their emotional, behavioral, and attitudinal responses within the UK age-verification context.

## Method

### Participants

Participants were recruited in the UK through the undergraduate participant pool in the School of Psychology at the University of Southampton and via Prolific, a research-oriented online platform. Eligibility was limited to individuals aged 18–25 who self-identified as male or female, reported having used pornography in the past year, and who lived in the UK.

### Procedure

An online survey was administered between October and December 2025. Participants were recruited through two sources: the undergraduate participant pool at the University of Southampton and Prolific. The study was advertised as an online survey about young adults’ pornography use and views of the UK age-verification requirements for online pornography. The same eligibility criteria and online survey procedure were applied across both recruitment sources, with all participants completing the survey through Qualtrics. Participants first accessed an online information sheet outlining the purpose of the study, the voluntary nature of participation, and data confidentiality. They were then asked to provide informed consent electronically before proceeding to the questionnaire, which included both closed-ended measures and open-ended questions about pornography use and views of the UK age-verification requirements for online pornography. The study did not include a direct measure of individual exposure to age-verification procedures (e.g., frequency or type of verification encountered); the regulatory context was used to situate the study, rather than as an indicator of participants’ individual exposure to age-verification procedures. Qualtrics-based consistency and bot-detection checks were applied, and only responses that passed these checks were recorded for analysis. Participants recruited via SONA received modest course credits, and Prolific participants received £1.

### Measures

#### Demographics

Participants reported gender, age, ethnicity, sexual orientation, relationship status, the importance of religion in their everyday lives, and education/employment.

#### Pornography Use

At the beginning of this section, all participants were asked whether they had used pornography during the previous year, and only those who responded “Yes” proceeded to the subsequent questions. Participants were instructed to respond with reference to “*since 25 July 2025 (when the new age-verification regulations for online pornography came into effect)*”.

#### Frequency of Pornography use Patterns

Following Wu and Zheng (2022), participants indicated how often “*since 25 July 2025”* they used pornography in each context: (1) secret solitary use, (2) partner-aware solitary use, and (3) shared use; an individual could report multiple use patterns. Response options ranged from 1 (*Never*) to 8 (*Every day or almost every day*).

#### Type Preference of Pornography

Participants indicated their preference for ten types of pornography content with the item “*I like to watch… (e.g. Vaginal intercourse*,* Group sex (one man/top*,* more women/bottoms)*,* Group sex (one woman/bottom*,* more men/tops)*,* Softcore Pornography (not as sexually explicit as the hardcore type))*” Response options ranged from 1 (*Not at all*) to 7 (*Extremely*).

#### Sexual Shame

Sexual shame was evaluated using ten items adapted from the Sexual Shame Inventory (Seebeck, 2021; α = 0.76–0.86), designed to measure three dimensions: Sexual Inferiority (e.g., “*I worry people will find out about my sexual flaws.*”), Relational Sexual Shame (e.g., “*I feel ashamed to talk to others about my sexuality/sexual experiences.*”) and Internalized Sexual Shame (e.g., “*I replay sexual experiences I am ashamed of over and over in my mind*”). Responses range from 1 (*Strongly Disagree*) to 7 (*Strongly Agree*). Higher mean scores on each subscale indicate greater shame.

#### Open Questions about the Age Verification Law

Participants’ perspectives on, and responses to, the UK age-verification law for online pornography were assessed using five open-ended questions. These items were designed to elicit perceived emotional, behavioral, and attitudinal responses to the implementation of the restriction:

#### Perceived Intentions and Beneficiaries

*“Do you believe the intention behind this new restriction policy (i.e., the age-verification requirement for accessing online pornography) is well-meaning? Who do you think benefits from it? Please explain.”*

#### Impact on Sexual Behavior

*“Has this restriction affected your sex life or sexual behaviors in any way? If yes, please describe how and to what extent.”*

#### Emotional and Self-Perceptual Impact

*“Have you experienced any changes in your emotions, attitudes, or self-perceptions related to sexuality (e.g., sexual shame, anxiety) in response to this restriction? Please elaborate.”*

#### Overall Evaluation of the Restriction

*“What are your personal views on this restriction? Please share your thoughts*,* feelings*,* or concerns.*”

#### Bypass Intentions and Strategies

*“Have you ever considered or attempted to bypass the age-verification restriction? If yes*,* please share your thoughts*,* motivations*,* experiences*,* or strategies.*”

### Statistical Analyses

We employed a convergent mixed-methods design, combining descriptive quantitative analyses of structured data with qualitative analysis of open-ended questions.

Quantitative analyses were conducted in R (version 4.5.2) using the tidyverse and psych packages. Descriptive statistics were calculated for demographic characteristics, pornography use patterns, pornography content preferences, and sexual shame. Categorical variables are reported as frequencies and percentages. Continuous variables are reported as means and standard deviations. Quantitative findings were used to characterise the sample and to provide contextual background for the qualitative findings.

Qualitative data from the five open-ended survey questions were analyzed using Reflexive Thematic Analysis (RTA) following Braun and Clarke’s (2021) six-phase framework. RTA was selected for its flexibility, its compatibility with an exploratory mixed-methods design, and its suitability for examining sensitive and subjective accounts related to sexuality. All responses were read repeatedly to support familiarization with the dataset. Initial codes were generated inductively, capturing salient semantic and interpretative features across participants’ accounts. Coding was conducted across the dataset rather than on a question-by-question basis. Although the survey comprised more than one open-ended item, themes were not developed from individual questions; instead, patterns of meaning were identified across responses where conceptually relevant. Codes were iteratively reviewed and clustered into candidate themes, which were refined through ongoing comparison to ensure internal coherence and clear analytic distinctions. Reflexive notes were used throughout the process to document analytic decisions and to situate the researchers’ interpretative role. Qualitative findings were subsequently considered alongside quantitative results within the mixed-methods design.

### Researcher Reflexivity

The qualitative analysis was conducted by the first author, trained in health psychology, with prior experience analyzing sensitive topics related to sexuality and pornography use. Throughout the analytic process, attention was given to how personal assumptions about pornography, regulation, and privacy might shape interpretation. Reflexive memos were used to document analytic decisions and to distinguish participants’ accounts from the researcher’s own evaluative positions. Particular care was taken to interpret responses in descriptive terms, prioritizing participants’ expressed meanings and experiences. This reflexive stance informed theme development across all stages of analysis.

## Results

### Participants

Overall, 623 participants were retained in the final analytic sample. Three participants were excluded because they did not meet the inclusion criteria. Participants had a mean age of 21.4 years (SD = 2.31). The sample comprised 204 [32.7%] men and 419 [67.3%] women. Descriptive characteristics of the sample are presented in Table 1. The sample was predominantly White, and most participants identified as heterosexual or bisexual. Participants generally reported low religious importance and were most commonly students, either exclusively or in combination with paid work. Relationship status, educational or employment status, ethnicity, sexual orientation, and religious importance are reported in Table 1. The survey used required-response settings for closed-ended items, so there was no item-level missingness for the descriptive quantitative measures reported below. Open-ended responses varied in length and detail, but all participants provided usable information for at least part of the survey; therefore, no participants were excluded because of missing or limited open-ended text.

**Table 1:** Participant characteristics

		*N*	%
Gender	Men	204	32.7
	Women	419	67.3
Ethnicity	White	438	70.3
	Black/African/Caribbean	38	6.1
	Asian	96	15.4
	Mixed (2 + groups)	34	5.5
	Other	7	1.1
	Prefer not to say	10	1.6
Sexual orientation	Heterosexual/straight	395	63.4
	Gay/lesbian	56	9.0
	Bisexual	151	24.2
	Asexual	3	0.5
	Prefer not to say	9	1.4
	Other	9	1.4
Relationship status	Single	251	40.3
	Dating	115	18.5
	Committed (not cohabiting)	173	27.8
	Married/Cohabiting	77	12.4
	Other	7	1.1
Education/employment	Working (FT/PT)	185	29.7
	Student	311	49.9
	Working & Student	81	13.0
	Unemployed	44	7.1
	Stay home parent	2	0.3
Religious importance	1 (Not at all)	358	57.5
	2	97	15.6
	3	49	7.9
	4	51	8.2
	5	33	5.3
	6	21	3.4
	7 (Very Important)	14	2.2

### Descriptive Quantitative Findings

#### Frequency of Pornography use Patterns

All participants included in the analytic sample had reported pornography use in the previous year as part of the study inclusion criteria. Descriptive results therefore refer to the frequency of specific pornography use contexts since 25 July 2025, rather than to lifetime or past-year pornography use overall. Participants reported pornography use across secret solitary, partner-aware solitary, and shared-use contexts (see Table 2).

**Table 2:** Number and percentages of reported frequency of pornography use patterns

	Option	Single		In Relationship	
		*N* = 251	%	*N* = 372	%
Secret Solitary Use	Never	46	18.3	88	23.7
	No more than twice	10	4.0	36	9.7
	Once every 3 months	10	4.0	26	7.0
	Once every 2 months	10	4.0	15	4.0
	Once a month	32	12.8	43	11.6
	Once every two weeks	35	13.9	50	13.4
	Once a week or more	73	29.1	93	25.0
	Every day or almost every day	35	13.9	21	5.6
Partner Aware Solitary Use	Never	—	—	182	48.9
	No more than twice	—	—	37	9.9
	Once every 3 months	—	—	18	4.8
	Once every 2 months	—	—	13	3.5
	Once a month	—	—	38	10.2
	Once every two weeks	—	—	34	9.1
	Once a week or more	—	—	42	11.3
	Every day or almost every day	—	—	8	2.2
Shared Use	Never	—	—	278	74.7
	No more than twice	—	—	33	8.9
	Once every 3 months	—	—	12	3.2
	Once every 2 months	—	—	12	3.2
	Once a month	—	—	15	4.0
	Once every two weeks	—	—	11	3.0
	Once a week or more	—	—	9	2.4
	Every day or almost every day	—	—	2	0.5

Note. Partner-aware solitary use and shared use were assessed only among participants in a relationship; cells not applicable to single participants are indicated by dashes (—)

Among single participants, secret solitary use was the only pornography use context assessed, as partner-aware solitary use and shared use were only applicable to participants in a relationship. Approximately one third reported using pornography secretly and alone once a week or more since 25 July 2025, whereas less than one-fifth reported no secret solitary use during this period.

Among participants in a relationship, nearly one quarter reported no secret solitary use since 25 July 2025, whereas approximately one third reported secret solitary use once a week or more. Partner-aware solitary use was less frequently reported than secret solitary use. Nearly half of partnered participants reported no partner-aware solitary use since 25 July 2025, while 13.5% reported this pattern once a week or more. Shared pornography use was the least frequently reported context. Three quarters of partnered participants reported no shared use since 25 July 2025, and only 2.9% reported shared use once a week or more.

Across the descriptive mean frequency scores, men reported higher average scores than women for each assessed pornography use context within both relationship-status groups (see Supplementary Table A).

#### Pornography Content Preferences

Participants’ preferences for the ten pre-specified pornography content categories are summarized in Table 3. These results describe reported preferences only and should not be interpreted as indicating content availability, actual access, or changes following the implementation of the UK age-verification requirements. Descriptively, the highest mean preference ratings were observed for vaginal intercourse, amateur content, oral sex, and softcore pornography. Lower mean ratings were observed for anal intercourse, group-sex categories, BDSM, gay/lesbian/queer content, and professional/mainstream content (see Supplementary Table B).

**Table 3:** Number and percentages of reported pornography content preferences

	Option	Men		Women	
		*N*	%	*N*	%
Vaginal Sex Preference	1 (Not at all)	22	10.8	64	15.3
	2	8	3.9	20	4.8
	3	9	4.4	31	7.4
	4	33	16.2	89	21.2
	5	29	14.2	82	19.6
	6	48	23.5	80	19.1
	7 (Very Much)	55	27.0	53	12.6
Anal Sex Preference	1 (Not at all)	44	21.6	268	64.0
	2	48	23.5	51	12.2
	3	32	15.6	35	8.4
	4	22	10.8	31	7.4
	5	19	9.3	18	4.3
	6	17	8.3	10	2.4
	7 (Very Much)	22	10.8	6	1.4
Oral Sex Preference	1 (Not at all)	27	13.2	117	27.9
	2	10	4.9	48	11.5
	3	11	5.4	46	11.0
	4	48	23.5	85	20.3
	5	44	21.6	57	13.6
	6	28	13.7	43	10.3
	7 (Very Much)	36	17.6	23	5.5
Gay/Lesbian/Queer Content Preference	1 (Not at all)	99	48.5	153	36.5
	2	26	12.7	37	8.8
	3	15	7.4	36	8.6
	4	11	5.4	56	13.4
	5	17	8.3	54	12.9
	6	15	7.4	41	9.8
	7 (Very Much)	21	10.3	42	10.0
Group Sex (One Man/Top, Multiple Women/Bottoms) Preference	1 (Not at all)	72	35.3	195	46.5
	2	26	12.7	59	14.1
	3	30	14.7	48	11.5
	4	27	13.2	46	11.0
	5	27	13.2	33	7.9
	6	13	6.4	28	6.7
	7 (Very Much)	9	4.4	10	2.4
Group Sex (One Woman/Bottom, Multiple Men/Tops) Preference	1 (Not at all)	86	42.2	194	46.3
	2	44	21.6	49	11.7
	3	12	5.9	34	8.1
	4	22	10.8	51	12.2
	5	19	9.3	42	10.0
	6	13	6.4	31	7.4
	7 (Very Much)	8	3.9	18	4.3
BDSM Preference	1 (Not at all)	100	49.0	189	45.1
	2	24	11.8	70	16.7
	3	27	13.2	35	8.4
	4	21	10.3	47	11.2
	5	18	8.8	35	8.4
	6	10	4.9	21	5.0
	7 (Very Much)	4	2.0	22	5.3
Professional Content Preference	1 (Not at all)	43	21.1	161	38.4
	2	27	13.2	69	16.5
	3	31	15.2	55	13.1
	4	51	25.0	76	18.1
	5	28	13.7	40	9.5
	6	17	8.3	10	2.4
	7 (Very Much)	7	3.4	8	1.9
Amateur Content Preference	1 (Not at all)	20	9.8	114	27.2
	2	13	6.4	52	12.4
	3	13	6.4	46	11.0
	4	32	15.7	65	15.5
	5	59	28.9	56	13.4
	6	36	17.6	48	11.5
	7 (Very Much)	31	15.2	38	9.1
Softcore Content Preference	1 (Not at all)	33	16.2	87	20.8
	2	30	14.7	47	11.2
	3	20	9.8	55	13.1
	4	48	23.5	94	22.4
	5	39	19.1	70	16.7
	6	26	12.7	33	7.9
	7 (Very Much)	8	3.9	33	7.9

#### Sexual Shame

All participants had reported pornography use in the past year as part of the study eligibility criteria. The grouping shown in Supplementary Table C distinguishes participants according to whether they reported pornography use since 25 July 2025 and should not be interpreted as indicating cessation or policy-related change. Among participants who reported pornography use since 25 July 2025, mean scores were 10.16 (SD = 4.98) for sexual inferiority, 11.26 (SD = 5.76) for relational sexual shame, and 7.41 (SD = 4.40) for internalized sexual shame. Among participants who did not report pornography use since 25 July 2025, mean scores were 10.72 (SD = 5.12) for sexual inferiority, 11.11 (SD = 5.24) for relational sexual shame, and 7.63 (SD = 4.43) for internalized sexual shame.

### Qualitative Findings: Young Adults’ Interpretations and Responses to the Age-Verification Policy

The qualitative analysis identified four overarching themes capturing how participants interpreted and responded to the introduction of age-verification requirements. These themes are presented alongside the descriptive quantitative findings to contextualize participants’ accounts of pornography use, privacy, emotional responses, and views on age verification.

#### Theme 1: Reframing the Social Meaning of Pornography Access

Participants described the age-verification requirement not primarily as a technical restriction on access, but as a policy that altered the social meaning of accessing pornography. Rather than focusing on whether access was possible, participants framed the policy as reshaping how pornography use was positioned and understood. These accounts centered on symbolic meanings attached to access, particularly in relation to personal identity, perceptions of observation, moral signaling, and concerns about data vulnerability.

#### Identity-Linking

Some participants described the policy as transforming pornography access from an anonymous activity into one perceived as personally identifiable. This shift was articulated through references to facial verification and the requirement to upload official identification, framing access as explicitly linked to the individual. One participant reported feeling “annoyed” at “having to show my face to get verified” (M, 25), while another contrasted prior anonymity with the current situation, stating that “it used to feel okay because it’s anonymous, but it feels like I can be identified now” (W, 20)^2^. Similarly, the requirement to submit identification materials was described as intrusive, with one participant referring to “having to upload an image of your ID” as “a breach of privacy” (W, 20).

#### Surveillance Concern

Some participants framed the policy in terms of a heightened sense of being observed. While these accounts reflected subjective perceptions rather than claims of actual monitoring, describing how the policy was interpreted as introducing a sense of observation into an activity that participants previously understood as private. One participant stated that it “felt like I was being watched and monitored whenever I try to access pornographic content” (W, 20), while another reported, “I have started feeling like I am under surveillance more” (W, 19). More generally, participants referred to “increasing surveillance of online activities” as “slightly concerning” (W, 20).

#### Moral Signaling

Participants also described the policy as conveying a moral message about pornography use. In these accounts, age verification functioned as a cue that framed access as improper, taboo, or socially disapproved. One participant described access as feeling “like I’m doing something I shouldn’t, like it’s a dirty behavior” (W, 18), while another stated that the restrictions “make it feel like you shouldn’t be accessing and using it” (W, 18). Similarly, encountering verification prompts was described as “somewhat shameful” (M, 24). Social judgement was also implied, with participants expressing concern that they might be “looked down on” (M, 21). Beyond individual experience, the policy was perceived as communicating broader norms, with one participant noting that it “gives the impression that sex is dirty and forbidden to young people” (W, 20).

#### Data Vulnerability and Hacking Concerns

Some participants framed the policy as introducing risks associated with identity verification, including potential exposure to hacking, data leaks, or blackmail, thereby reframing pornography access as an activity carrying risks beyond the act itself. One participant described the policy as “open[ing] a bunch of people up to being blackmailed after the verification data ends up getting leaked,” characterizing this as a misunderstanding of “how the internet works” (M, 25). Others expressed similar concerns, noting that “hackers may gain access to emails, names or other information” (W, 25). Relatedly, one participant stated that they did not agree with the “methods of data collection due to cyber security issues,” describing pornography access as “an already private activity” that could be “hacked and used as blackmail” (W, 22).

#### Theme 2: Reported Behavioral Adjustments to Accessing Online Pornography Under Age-Verification Requirements

Participants described a range of practical adjustments in how they accessed online pornography following the introduction of age-verification requirements, reflecting different ways of responding to the new access conditions, including withdrawing from verified platforms, relocating access to alternative platforms, reducing use, and actively bypassing verification systems.

#### Avoidance of Age-Verified Sites and Shifting Access Pathways

Some participants described avoiding or disengaging from specific platforms that required age verification. This involved choosing not to proceed once verification was requested, leaving sites immediately, or selectively avoiding platforms that introduced verification measures. One participant stated, “I no longer use [mainstream porn site] due to the age verification” (M, 20), while another noted, “I just don’t go on sites that ask me to verify anymore” (W, 20). Similar responses included “If a site asks for ID, I just leave” (W, 20) and “I stopped using those sites once they brought in verification” (W, 24). Alongside avoiding age-verified sites, some participants described relocating where they accessed pornographic content rather than stopping use. This included shifting away from mainstream pornography websites toward alternative platforms that did not require age verification, such as social media platforms (e.g., Reddit, X). One participant reported, “I mainly use [social media] now instead” (W, 20), while another stated, “I don’t use porn sites anymore, I just see content on [social media]” (W, 19). Similar shifts were described as switching to “platforms that don’t require verification” (M, 23) or looking for “the same content on social media instead” (M, 24).

#### Reduction

Some participants reported a reduction in use rather than complete disengagement. In these accounts, pornography remained accessible in principle but was used less frequently due to the perceived inconvenience or effort associated with age-verification requirements. Participants described watching pornography “less now because it feels like too much effort” (W, 20) or thinking about accessing content but “not bother[ing] going through the process” (W, 18). Others stated that they used pornography “far less often since the checks were introduced” (W, 23) or that they “sometimes” decided “it’s not worth it” (M, 19). Notably, all participants quoted in this subsection also described shame, discomfort, or reluctance when attempting to access pornography (see Theme 3), although these accounts are reported as being participants’ subjective narratives rather than evidence of a causal pathway.

#### Circumvention

A further set of responses involved actively bypassing age-verification systems. Participants described using technical workarounds to access content without completing verification. These included statements such as “I just use a VPN” (W, 22), “Changing my location with a VPN works” (W, 20), and “I access it through sites based outside the UK” (W, 20). Others described VPN use as “the easiest way around it” (W, 19).

#### Theme 3: Differentiated Psychological Experiences following Policy Implementation

Participants described a range of emotional and self-perceptual experiences in relation to the age-verification requirements, including little to no change and feelings of shame, anxiety, confidence, or calm.

#### Experiences of Shame or Anxiety

Whereas earlier analyses focused on how the policy was understood as conveying social or moral meanings, this theme examines participants’ reported emotional experiences. Some participants reported emotional experiences characterized by heightened feelings of shame or anxiety when engaging with or attempting to access pornographic content. One participant stated, “I now feel ashamed if I access porn, as I feel like I shouldn’t” (W, 21), while another reported that the policy “made me feel more shameful and more anxious” (W,19). Others similarly described access as feeling “a bit more shameful” than before (M, 21).

#### Experiences of Reduced Pressure or Emotional Ease

In contrast, other participants described experiences marked by reduced pressure, greater ease, or shifts in sexual self-perception. One participant reported feeling “better about myself sexually, as I’m not exposed to unrealistic standards as often” (M, 18), while another described feeling “more confident and less pressured than before” (W, 19). Others referred to feeling “calmer and less focused on sexual content” (W, 22). Some participants linked these experiences to reduced comparison, stating that their “overall sexual shame has lessened because I am not constantly comparing myself to pornstars” (W, 23). Notably, accounts within this subtheme were provided by participants who had reported pornography use in the previous year but did not report pornography use since 25 July 2025; this grouping should not be interpreted as indicating cessation or policy-related change.

#### Little to no Emotional Change

Many participants reported little to no noticeable emotional change following the introduction of age verification. These responses were often framed in terms of pornography not occupying a central role in their lives. One participant stated that the policy had “no effect on my attitudes, self-perceptions, or emotions” (M, 20), while another reported, “No, none. I don’t see an issue with pornography, and this hasn’t changed anything for me” (W, 18). Similar responses included “No, it hasn’t affected my emotions, attitudes or self-perceptions” (W, 18) and “Not really, it’s not something that plays a big role in my life” (W, 19).

#### Theme 4: Perceived Policy Legitimacy and Alternative Governance

Participants reported varied evaluations of the age-verification policy’s legitimacy, effectiveness, and appropriate scope. Accounts reflected multiple ways of assessing whether the policy was justified, whom it served, and whether alternative forms of governance might be more appropriate.

#### Well-Intentioned but Ineffective

A common position framed the policy as motivated by concern for protecting children or under-18s while simultaneously questioning its practical effectiveness. Participants acknowledged the stated intent but emphasized the ease with which restrictions could be bypassed. One participant noted that “if the pure intent is to ensure young adults don’t access pornography, then it seems well intended,” while adding that “the likelihood of children finding different ways to access 18 + material is extremely likely” (M, 25). Others similarly described the policy as “well-meaning” but may be ineffective, noting that “children will still find ways around it” (W, 21) or that it “doesn’t really stop people who want to access it” (W, 22).

#### Collateral Harm and Privacy Concerns

Some participants questioned the policy’s legitimacy on the grounds that it imposed unnecessary burden on adults. These concerns centered on privacy, data handling, and perceived monitoring. One participant expressed concern that the policy could lead to the government “tracking or monitoring what individuals over the age of 18 watch” (W, 19), while another described age verification as “forcing people to put their data online,” characterizing it as “a disaster waiting to happen” (M, 25). Others similarly referred to the policy as raising “unnecessary privacy concerns for adults” (M, 20).

#### Symbolic Governance

Some participants interpreted the policy as symbolic rather than as a practical intervention. From this perspective, the restriction was seen as signaling action without addressing underlying issues. One participant suggested that “the government benefits from it the most,” as it “makes them feel like they’ve done something good” (W, 23). Others described the policy as “more like a symbolic gesture than something that actually fixes the problem” (W, 19) or stated that it “doesn’t really address the root issue” (W, 18).

#### Alternative forms of governance

Alongside critique, participants also raised alternative approaches they viewed as more appropriate. These centered on education and parental involvement rather than blanket restrictions. One participant argued that “parents should be able to block their children from seeing the content rather than the government blocking everyone” (W, 18), while others suggested authorities “should focus more on education rather than restrictions” (W, 18) or that “better sex education would be more effective than bans” (W, 19). Similar views emphasized the need to “start at the root of the problem and target education instead” (W, 20).

#### Explicit Endorsement of the Policy

In contrast, some participants expressed clear support for the policy, framing it as a justified safeguarding measure for children, particularly boys. Participants described the policy as “quite a good decision (for ‘young people’ and ‘boys’)” (W, 20), “good because access to pornography was far too easy before (for ‘children’)” (M, 18), and “a positive safeguarding action (for ‘children’)” (W, 19). Others explicitly stated that the restriction was “a good thing” and hoped that it would “stay in place (for ‘young men’ and ‘boys’)” (W, 20).

## Discussion

Age-verification requirements are often evaluated in terms of how effectively they restrict minors’ access to online pornography. However, this access-focused framing can overlook how legal-age users interpret and navigate these systems once they are introduced. In this study, young adults’ accounts showed that age verification was not experienced only as a technical check but also through concerns about privacy, identifiability, stigma, emotional response, circumvention, and policy legitimacy.

### Beyond Access: How Age Verification Reshapes Pornography Use and Meaning Among Young Adults

#### Reported Access Practices Under Regulatory Constraint

Young adults described varied ways of navigating pornography access under-age verification requirements. Their accounts included avoidance of age-verified sites, movement toward alternative platforms, reduced use, and circumvention. These access practices were often described alongside concerns about privacy, identifiability, and traceability. Some participants described verification as making access feel personally identifiable, traceable, or less anonymous. This aligns with the surveillance perspective, in which digital access systems can make private activities feel subject to identification, monitoring, or data capture. Such concerns may be especially relevant in relational contexts, where pornography use can involve negotiation between privacy, disclosure, and partner awareness (Li et al., 2026).

Circumvention was one practical response described by participants, including the use of VPNs or non-UK sites to bypass verification. Notably, references to VPN use were especially prominent and align with reports of increased VPN uptake following the introduction of comparable policies (Maris et al., 2020). Following the implementation of the Online Safety Act in July 2025, VPN registrations reportedly increased by over “1,400%” (Electronic Frontier Foundation, 2025). Although the present study cannot establish how common these practices are beyond the sample, participants’ accounts suggest that age-verification requirements may be experienced by some young adults as redirecting access rather than simply preventing it. This raises a policy concern: users who avoid verified commercial sites may seek content on platforms where regulatory oversight, content moderation, and user protections are less closely monitored.

#### Divergent Psychological Responses to Regulation

Participants’ qualitative accounts indicated varied psychological responses to age-verification requirements. Some described little emotional impact, whereas others reported shame, anxiety, discomfort, reduced pressure, or greater emotional ease in relation to pornography access. These accounts clearly indicate that age verification was not experienced in a uniform way.

Some participants who reported reduced pornography use described heightened shame associated with the verification process. For instance, participants described accessing pornography “less now because it feels like too much effort” (W, 20), whilst noting, “I feel slightly more sexual shame surrounding online pornography, which also impacts my decision to engage with it” (W, 20). Others reported feeling “more shameful about using pornography and more anxious about using it” (W, 18) or stated, “It feels a bit more shameful to access porn now, even on legal websites. Having to use a VPN feels like I’m breaking the rules despite being over 18” (M, 21). These accounts suggest that verification requirements may make pornography access feel more salient or morally charged for some individuals, particularly when access is already experienced ambivalently. A stigma perspective helps clarify why this salience may be emotionally consequential: when a private behavior is felt to be exposed to social judgement or moral disapproval, shame may become more prominent. This interpretation is consistent with research on moral incongruence, which shows that distress related to pornography use often arises from discrepancies between behavior and personal values rather than from use itself (Bőthe et al., 2022). In this context, verification requirements may be experienced as signaling social disapproval while also introducing anxieties around data exposure and rule-breaking (Borgogna et al. 2024; Li et al. 2025b).

A subset of participants who did not report pornography use since 25 July 2025 described positive psychological experiences, including feeling “more confident, less stressed”. These individuals explicitly linked improved well-being to less exposure to “unrealistic standards” and reduced social comparison with pornography performers. This pattern aligns with substantial evidence that pornography consumption, particularly amongst women, is associated with body image concerns and performance anxiety during sexual activity (Demirgül et al. 2025; Li et al. 2025a; Litsou et al. 2024). For these participants, not reporting pornography use during the study recall window was described as emotionally beneficial, but this should not be interpreted as evidence that the policy directly led to cessation or improved well-being.

Taken together, the qualitative accounts suggest that shame-related responses may depend on how participants already understand pornography use, privacy, moral judgement, and self-comparison. Rather than suggesting a generalized change in sexual shame, the findings point to a range of subjective experiences within the age-verification context (Borgogna et al., 2024; Droubay et al., 2024).

### How Young Adults Evaluate and Make Sense of the Policy

#### When Regulation Communicates Values as well as Rules: Surveillance, Morality, and the Internalisation of Policy Messages

Some participants interpreted age verification not merely as a mechanism of access control, but as a form of symbolic communication conveying social values about pornography use. Participants described the policy as signaling that pornography consumption is “dirty” or “shameful”, framing verification as a moral judgement rather than a neutral protective measure. Viewed through a stigma perspective, these accounts suggest that identity verification may make a private, legal sexual behavior feel more visible to possible judgement. Notably, this differed from how age restrictions may operate in other domains, such as alcohol consumption or driving, where reaching the required age can be understood as a recognized marker of adulthood. In contrast, some participants described pornography age verification as embarrassing or stigmatizing, suggesting that it may reinforce rather than reduce the sense that pornography use is socially judged (Macdowall et al., 2025). By associating access to legal sexual content with identity verification, participants sometimes interpreted the policy as implying that pornography use was exceptional, suspicious, or worthy of monitoring in ways that other legal online activities are not (Moss, 2025). Such symbolic cues may be particularly salient for young adults, a life stage characterized by ongoing development of sexual identity and sexual self-concept, during which individuals more actively negotiate and internalize sexual norms within broader social and cultural contexts (Bishop et al., 2024; Jastrzębska & Błażek, 2022).

Participants’ surveillance-related concerns warrant particular attention. Young adults’ descriptions of feeling “watched” or “monitored” do not necessarily reflect technical understanding of actual surveillance; rather, they reflect perceived surveillance and concerns about privacy, visibility, and observation. This interpretation is consistent with a surveillance perspective, which draws attention to how digital systems can make personal activities feel subject to identification, data collection, and/or monitoring. Data governance and tracking issues on adult content platforms have faced sustained scrutiny in recent years; media reporting on pornography platform tracking indicates users may remain unclear about how their data are collected, inferred, and shared (Burgess, 2023). Meanwhile, recent research on digital privacy and pornography consumption found that “93%” of pornography websites leak data to third parties (Maris et al., 2020, p. 2025). Against this backdrop, participants’ concerns about verification data being “leaked”, “hacked”, or used for “blackmail” can be understood within wider concerns about data vulnerability in online sexual contexts. Findings reflect the perceived psychological burden that may accompany requests for sensitive identity credentials in relation to a stigmatized activity.

Moreover, social evaluations of sexual behavior continue to reflect a sexual double standard, whereby comparable sexual activities are more likely to attract stricter moral scrutiny and negative judgment when associated with women, resulting in higher reputational and social costs (Gómez-Berrocal et al., 2022). Within this social context, women’s pornography use, if perceived as disclosed, may be subject to particularly intense normative scrutiny. Previous qualitative research similarly suggests that women may describe pornography use in terms of both pleasure and distress, alongside sensitivity to social evaluation and perceived consequences (Litsou et al., 2024). Consequently, when age verification binds pornography access to personally identifiable information, the resulting sense of visibility may not be experienced as gender-neutral. For some women, exposure risks may carry particular subjective weight, given broader evidence that women’s sexual behavior is often subject to heightened social evaluation (Endendijk et al., 2020; Jarvie & Renaud, 2024).

#### Who Supports, who Resists, and why

Participants articulated divergent evaluations of the age-verification policy, reflecting multiple ways of assessing its intent, effectiveness, and legitimacy.

A substantial proportion acknowledged the policy’s protective intent, framing it as a “well-intentioned” measure aimed at preventing minors’ access. Some participants explicitly supported the policy, describing it as “a good decision” or a “positive safeguarding measure”. Notably, these supportive accounts often focused on protecting children, rather than on personal benefit, suggesting that although some adult participants experienced the policy as burdensome or ineffective for themselves, some nevertheless endorsed it as a broader social intervention. For example, one participant emphasized that contemporary young people are “growing up too fast” and suggested that age verification “shields them from something they will organically grow into at a certain age” (W, 23), protecting them from premature exposure to content that should be gradually understood at more mature stages. Such support, however, was rarely unqualified. Many of these same participants expressed concern that the policy affects not only children but “everyone”, acknowledging that its burdens fall disproportionately on adults and questioning whether minors might still circumvent restrictions. Doubts about effectiveness were further reflected in participants’ own reported access practices. Those who reported using VPNs, migrating to unregulated platforms, or accessing content via social media described ways in which age-verification requirements could be bypassed or avoided. Some circumvention was not framed as deviant or exceptional but straightforwardly: “I just use a VPN”, which is “the easiest way to get around it”. This direct and matter-of-fact tone also indirectly reflects the potential normalization and accessibility of such circumvention behaviors.

Some participants framed the policy as a form of “symbolic governance”, questioning it as a measure that benefits the government by signaling action while failing to address underlying issues. These interpretations resonate with recent research documenting public skepticism regarding the effectiveness of age-restriction measures and concerns that such policies priorities symbolic reassurance over substantive impact (Jarvie & Renaud, 2024; Moss, 2025). Evidence from multiple jurisdictions has raised concerns about circumvention and limited effects on minors’ actual access, particularly given that younger generations are often more technically adept than policymakers assume (and even the policymakers themselves) (Internet Matters, 2025). Additionally, participants’ proposed alternative governance approaches merit attention. They advocated education-based interventions from parents or schools rather than access restrictions, believing “better sex education would be more effective than bans”. These evaluations also speak to wider debates about responsibility for age verification. One issue concerns whether verification should be conducted by pornography providers themselves or outsourced to third-party verification services. Third-party systems may reduce the amount of sensitive identity information held directly by pornography providers, but they may also create uncertainty about who collects, stores, verifies, and safeguards users’ data. Participants’ concerns about privacy, data leaks, and trust therefore point to a broader question of accountability in the governance of online pornography access. This suggestion aligns with public health scholarship emphasizing the value of comprehensive sexuality education over abstinence-based approaches (Rodríguez-García et al., 2025; Versloot-Swildens et al., 2024).

### Implications, Limitations and Future Research

This study is among the earliest empirical investigations to examine young adults’ subjective experiences of online pornography age-verification policies, thereby foregrounding a critical user perspective. The mixed-methods design enabled us to situate participants’ open-ended accounts alongside descriptive information about pornography use contexts, content preferences, and sexual shame, offering a more comprehensive account than either approach alone. The findings highlight how young adults may interpret age-verification requirements in relation to privacy, surveillance, stigma, access practices, and policy legitimacy.

Meanwhile, several limitations should be acknowledged. First, the absence of a direct measure of individual exposure to age-verification procedures means that findings reflect participants’ experiences within the broader regulatory context, rather than measured exposure to verification itself. Second, the sample did not include gender minority participants, limiting the generalizability of the findings. Their experiences may differ in relation to privacy, surveillance, disclosure, and identity verification, especially given that some groups are themselves subject to greater surveillance (and stigmatization) in their everyday activities (Scheim et al., 2020). Future research should employ longitudinal or panel designs and incorporate direct measures of exposure to age-verification processes to better assess behavioral and psychological change over time, as well as examining more diverse populations.

## Conclusion

Although age-verification requirements for online pornography are primarily designed around minors’ access, less is known about how these requirements are experienced by legal-age young adults. By examining young adults’ accounts within the UK age-verification context, this study shows that age verification was experienced by some young adults less as a neutral compliance step than as a shift in the meaning of access, one that made pornography use feel more traceable, more morally charged, and less private. Participants described varied access practices, including avoidance, migration to alternative platforms, reduced use, and circumvention, indicating that age-verification requirements may be experienced by some legal-age users as redirecting access. Emotional responses were similarly varied. Qualitative accounts described a diverse spectrum of shame, anxiety, relief, improved self-perception, and minimal emotional impact, pointing to heterogeneous experiences shaped by participants’ existing orientations toward pornography, privacy, and moral judgement. Participants’ general evaluations of the policy were similarly varied, combining child-protection rationales with concerns about privacy and “governance creep.” These findings suggest that access-focused regulation may be limited if it does not account for user trust, privacy concerns, and movement toward less regulated digital spaces. This tension between state intervention and individual liberty is reflected in participants’ own accounts, as illustrated by this observation: “Something like heavily age-restricting porn seems small but can snowball in the future and lead to further unreasonable restrictions, reducing expression and freedom of choice” (M, 20). For research on early adulthood, the findings show that digital sexual regulation may matter not only for access to sexual content, but also for how young adults negotiate privacy, visibility, sexual stigma, and autonomy during a developmental period in which sexual norms and identities remain actively shaped.

## Supplementary Information

Below is the link to the electronic supplementary material.


Supplementary Material 1


## Data Availability

This manuscript's data will not be deposited.

## Footnotes

1 Age categories in sexuality research are usually not fixed, and terms such as *adolescents* often encompass diverse legal and developmental stages. This study, therefore, uses *young adults* to refer specifically to participants aged 18–25, avoiding assumptions of homogeneity across broader youth categories.

2 In all quoted material, participant information is indicated in the format *(G*,* A)*, where *G* refers to reported gender (*M* = man; *W* = woman), and *A* refers to age in years. Because the study included only men and women aged 18–25, the same gender–age combination (e.g., *(W*,* 20)*) may appear more than once within a given theme or subtheme. However, each quotation represents a different participant, and individual participants are only quoted once within the same theme.

## Declarations

### Preregistration

This study was preregistered on OSF Registries on August 20, 2025. The preregistration included the study design, hypotheses, and planned target analyses. The preregistration is available at 10.17605/OSF.IO/AX6JW.
