# Supplementary material for: A Timely Exploratory Study of Young Adults’ Psychosexual Responses to the UK’s New Age Verification Law on Online Pornography
Source: J Youth Adolesc. 2026 Aug 3;55(9):2225–40. doi: 10.1007/s10964-026-02397-8 (PMC13534180; doi:10.1007/s10964-026-02397-8)
Supplement: Supplementary file 1 [file 10964_2026_2397_MOESM1_ESM.docx]

**Table A.**

*Descriptive statistics of reported frequency of pornography use patterns*

|  | Gender | Secret Solitary Use | | Partner Aware Solitary Use | | Shared Use | |
| --- | --- | --- | --- | --- | --- | --- | --- |
|  |  | *M* | *SD* | *M* | *SD* | *M* | *SD* |
| Single | Men (n=104) | 6.38 | 1.93 | — | — | — | — |
|  | Women (n=147) | 4.31 | 2.44 | — | — | — | — |
| In Relationship | Men (n=100) | 5.97 | 2.06 | 3.33 | 2.62 | 1.83 | 1.66 |
|  | Women (n=272) | 3.81 | 2.4 | 2.87 | 2.28 | 1.71 | 1.55 |
| *Note.* Participants indicated how often “since 25 July 2025” they used pornography in each context: (1) secret solitary use, (2) partner-aware solitary use, and (3) shared use; an individual could report multiple use patterns. Response options include 1 (Never), 2 (No more than twice in the past 3 months), 3 (Once in the past 3 months), 4 (Once in the past 2 months), 5 (Once a month), 6 (Once every two weeks), 7 (Once a week or more) and 8 (Every day or almost every day). | | | | | | | |

**Table B.**

*Descriptive statistics of reported pornography content preferences*

| Gender | Vaginal intercourse | | Anal intercourse | | Oral Sex | | Gay/Lesbian/Queer | | Group sex (one man/top) | |
| --- | --- | --- | --- | --- | --- | --- | --- | --- | --- | --- |
|  | *M* | *SD* | *M* | *SD* | *M* | *SD* | *M* | *SD* | *M* | *SD* |
| Men (n=204) | 4.98 | 1.94 | 2.8 | 1.73 | 4.58 | 2.09 | 2.75 | 2.17 | 2.93 | 1.87 |
| Women (n=419) | 4.33 | 1.91 | 1.74 | 1.35 | 3.27 | 1.99 | 3.27 | 2.15 | 2.49 | 1.78 |
|  | Group sex (one woman/bottom) | | BDSM | | Professional/Mainstream | | Amateur | | Softcore Pornography | |
|  | *M* | *SD* | *M* | *SD* | *M* | *SD* | *M* | *SD* | *M* | *SD* |
| Men (n=204) | 2.58 | 1.85 | 2.41 | 1.72 | 3.36 | 1.72 | 4.61 | 1.79 | 3.69 | 1.76 |
| Women (n=419) | 2.67 | 1.94 | 2.57 | 1.88 | 2.59 | 1.62 | 3.46 | 2.05 | 3.58 | 1.86 |

**Table C.**

*Descriptive statistics of sexual shame*

| Use Status | Gender | Sexual Inferiority | | Relational Sexual Shame | | Internalised Sexual Shame | |
| --- | --- | --- | --- | --- | --- | --- | --- |
|  |  | *M* | *SD* | *M* | *SD* | *M* | *SD* |
| Reported use since 25 July 2025 | Men (n=189) | 9.66 | 4.81 | 11.19 | 5.65 | 6.39 | 3.78 |
|  | Women (n=318) | 10.46 | 5.06 | 11.31 | 5.82 | 8.02 | 4.55 |
| No reported use since 25 July 2025 | Men (n=15) | 8.87 | 4.67 | 10.2 | 5.12 | 5.8 | 3.67 |
|  | Women (n=101) | 11 | 5.13 | 11.24 | 5.27 | 7.9 | 4.49 |
